# 3D LIDAR-Camera Extrinsic Calibration Using an Arbitrary Trihedron

**DOI:** 10.3390/sl30201902

**Published:** 2013-02-01

**Authors:** Xiaojin Gong, Ying Lin, Jilin Liu

**Affiliations:** Department of Information Science and Electronic Engineering, Zhejiang University, Hangzhou 310027, Zhejiang, China; E-Mails: ly86@zju.edu.cn (L.Y.); liujl@zju.edu.cn (J.L.)

**Keywords:** extrinsic calibration, 3D LIDAR-camera system, sensor fusion

## Abstract

This paper presents a novel way to address the extrinsic calibration problem for a system composed of a 3D LIDAR and a camera. The relative transformation between the two sensors is calibrated via a nonlinear least squares (NLS) problem, which is formulated in terms of the geometric constraints associated with a trihedral object. Precise initial estimates of NLS are obtained by dividing it into two sub-problems that are solved individually. With the precise initializations, the calibration parameters are further refined by iteratively optimizing the NLS problem. The algorithm is validated on both simulated and real data, as well as a 3D reconstruction application. Moreover, since the trihedral target used for calibration can be either orthogonal or not, it is very often present in structured environments, making the calibration convenient.

## 1. Introduction

Multi-sensors are commonly equipped on mobile robots for navigation tasks. Currently, for instance, ranging sensors such as high-speed 3D LIDARs are often used in conjunction with cameras for a robot to detect objects [1,2] and reconstruct scenes [3–5]. In these sensor fusion-based applications, a prerequisite is to extrinsically calibrate the relative transformation between the sensors. The result of extrinsic calibration highly impacts subsequent fusion processes.

A variety of methods have been developed to address the LIDAR-camera extrinsic calibration problem. Among them, early interest focuses on systems consisting of a 2D LIDAR and a camera [6-9]. Wasielewski and Strauss [6] and Naroditsky *et al.* [7] calibrate a 2D laser scanner with respect to a camera by making use of special calibration rigs, such as a white planar board covered with a black line. The work of Zhang and Pless [8] relies on a planar checkerboard pattern. Corners of the pattern [10] are first detected in images and used to determine the poses of planes in camera frames. Meanwhile, 3D points falling on the checkerboard are taken into consideration to estimate the planes' poses in LIDAR frames. Using the geometric constraint of the planar target in a couple of LIDAR-camera observations, the extrinsic calibration problem is formulated as a nonlinear least squares (NLS) problem [11] and solved iteratively.

In recent years, with the development of 3D laser ranging techniques, several methods were proposed to calibrate 3D LIDAR-camera systems [12–18]. Unnikrishnan [12] and Pandey *et al.* [13] extend the checkerboard pattern-based method [8] from 2D to 3D LIDARs. Mirzaei *et al.* [19] utilizes a planar board covered with fiducial markers for calibration, which, in essence, is of the same rationale as the checkerboard-based approaches. They further divide the NLS optimization problem into two least-square sub-problems and solve them analytically. The checkerboard pattern is also used in the work of Geiger *et al.* [20]. They calibrate a 3D LIDAR-camera system using a single shot containing such multiple patterns. Instead of using planar checkerboard patterns, there are several alternative methods that rely on correspondences of points [21], lines [22] or circles [14], or employ inertial sensors [23,24]. Compared to plane-based approaches, most of these methods need to build point- or line-wise correspondences between images and LIDAR points. However, due to the lower and non-uniform resolution of LIDAR measurements, it is difficult to achieve high accuracy.

In this work, we propose a novel way to conduct the extrinsic calibration between a 3D LIDAR and a camera. In contrast to most of the published techniques, our method distinguishes itself in two aspects:

1. It takes advantage of a trihedron—which may or may not be orthogonal—for calibration. Such trihedral targets are ubiquitous in both indoor and outdoor structured environments, such as two adjacent walls of a building together with the floor. Hence, it is quite convenient for a robot to collect data for calibration. Compared to the aforementioned calibration rigs, the trihedral configuration is less likely to be perturbed even under severe weather conditions, and is easier to be captured.
2. In contrast to these calibration-rig-based methods that require a user to specify both the region of a plane in 3D LIDAR and the corners in images, our method requires fewer user inputs. Only the region of each plane of the trihedron in the sensors' data is needed. Moreover, the precision of the manual inputs does not make much of a difference.

To present the proposed method, we organize the remainder of this paper as follows. In Section 2, we first describe the extrinsic calibration problem via taking advantage of an trihedral calibration rig, and introduce the associated geometric and motion constraints. Section 3 presents the entire calibration procedure. Experiments conducted on both simulations and real data are exhibited in Section 4, followed with conclusions in Section 5.

## 2. Problem Description

Let us formally define the problem of 3D LIDAR-camera extrinsic calibration. We are given a camera and a 3D LIDAR that are rigidly mounted with respect to each other. Both sensors are assumed to be pre-calibrated, meaning that their intrinsic parameters are known. A trihedron is observed synchronously by them. Our objective is to determine the relative transformation between the two sensors, by taking advantage of the constraints associated with the trihedron.

For the sake of clarity, in the remaining of this section, we introduce the related definitions and notations, together with the geometric and motion constraints established between the measurements of the two sensors.

### 2.1. Definitions and Notations

**Figure 1.**
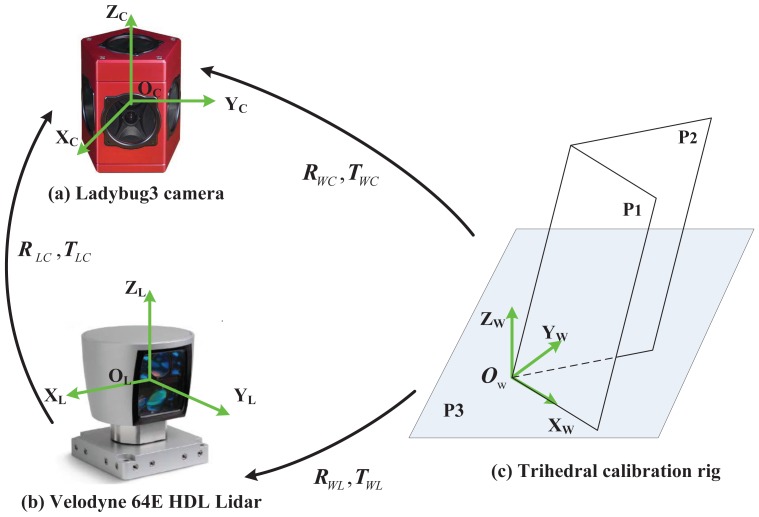
A typical calibration configuration. (**a**) is a Ladybug3 camera and (**b**) is a Velodyne HDL-64E LIDAR. Both are rigidly assembled with respect to each other. A trihedral object (**c**), which may or may not be orthogonal, is observed by both sensors.

Figure 1 demonstrates a typical calibration configuration. It includes a system composed of a Ladybug3 omnidirectional camera [25] and a commercially available high-speed Velodyne HDL-64E 3D LIDAR [26], as well as a trihedral target viewed by both sensors. In experiments, the trihedron is fixed and the sensor system moves to obtain multiple configurations. In such configurations, several reference frames are defined:

- **Camera frame:** The proposed method is not restricted to a specific camera type, as long as the camera is of a single viewpoint [27] and pre-calibrated. The camera frame is set up to be coincident with the one defined in its projection model. The frame is represented by {*C_i_*}, in which *i* = 1 ⋯ *N* indicates the *i^th^* configuration.
- **LIDAR frame:** The LIDAR frame is also defined to be coincident with the one in its own projection model, and is denoted by {*L_i_*}.
- **World reference frame:** The world reference frame is fixed on the trihedron. Since the trihedral object can be either orthogonal or not, the reference frame is set up in such a way that the origin is at the common vertex and the axis *X* aligns with one intersection line. The axis *Z* is aligned with the direction of the plane *P*_3_'s normal vector, and *Y* is further determined following the right-hand rule, as illustrated in Figure 1(c). The world frame is denoted by {*W*}.

Once the frames are defined, we represent the relative rotation and translation from one frame *A* to another frame *B* by **R***_AB_* and **T***_AB_*, where *A*, *B* ∈ {*C_i_*, *L_i_*, *W*}. Then, given a 3D point **P***_A_* in the frame A, the corresponding point **P***_B_* in **B** is computed via **P***_B_* = **R***_AB_***P***_A_* + **T***_AB_*. In practice, the sensors are rigidly mounted on a mobile robot, and the transformations from the LIDAR to the camera, *i.e.* **R***_L_i_C_i__* and **T***_L_i_C_i__*, are fixed in all configurations even when the robot moves. Hence, they are simply denoted by **R***_LC_* and **T***_LC_*, which are the parameters we aim to estimate in the calibration task.

In addition, we know that a plane in a frame is specified by **N***^T^***P** − *d* = 0, where **P** is an arbitrary 3D point lying on the plane, and **N** and *d* are, respectively, the normal vector and the distance. Hence, we use $\left\{{\text{N}}_{A}^{j},d_{A}^{j}\right\}$ to describe the *j^th^* plane of the trihedron with respect to the frame *A*, *j* = 1 ⋯ 3, and ${\text{P}}_{A}^{j,k}$ specifies the *k^th^* point on the plane.

### 2.2. Geometric and Motion Constraints

The proposed method makes use of a trihedron as a calibration rig. Hence, in order to address the extrinsic calibration problem, several constraints are taken into consideration. They are summarized as follows.

- **Trihedral constraint:** Let us consider the trihedron with respect to a sensor frame *A*. If its three planes, $\left\{{\text{N}}_{A}^{j},d_{A}^{j}\right\}$, are estimated, then the relative rotation **R***_WA_* and translation **T***_WA_* from the world frame to the sensor frame are uniquely determined. We represent **R***_WA_* = [**r***_WA_*1 **r***_WA_*2 **r***_WA_*3], where **r***_WA_*1, **r***_WA_*2 and **r***_WA_*3 are column vectors. Then, we have
  (1) $$\{\begin{array}{ll} {\text{r}}_{WA}3 & ={\text{N}}_{A}^{3}\\ {\text{r}}_{WA}1 & =\frac{{\text{N}}_{A}^{1}\times {\text{N}}_{A}^{3}}{\left\|{\text{N}}_{A}^{1}\times {\text{N}}_{A}^{3}\right\|}\\ {\text{r}}_{WA}2 & ={\text{r}}_{WA}3\times {\text{r}}_{WA}1 \end{array}$$
  and the translation is
  (2) $${\text{T}}_{WA}={\left[\begin{array}{ccc} {\text{N}}_{A}^{1} & {\text{N}}_{A}^{2} & {\text{N}}_{A}^{3} \end{array}\right]}^{-T}{\left[\begin{array}{ccc} d_{A}^{1} & d_{A}^{2} & d_{A}^{3} \end{array}\right]}^{T}$$
- **Planarity constraint between two frames:** This constraint implies that, if points in a frame *A* are coplanar, then they must lie on a plane when transformed to another frame *B*. It means that, in the absence of noise, we have a plane {*N_B_*, *d_B_*} such that
  (3) $$\sum_{k}{\left\|{\text{N}}_{B}^{T}\left({\text{R}}_{AB}{\text{P}}_{A}^{k}+{\text{T}}_{AB}\right)-d_{B}\right\|}^{2}=0$$
  for all coplanar points ${\text{P}}_{A}^{k}$ in the frame A.
- **Planarity constraint between two images:** This constraint describes the relationship between a set of coplanar feature points and their correspondences in two images. Given a single-viewpoint camera, for the purpose of generality, we represent its projection model as **p** = **F**(**P**), where **P** is a 3D point in space and **p** is the projected image point. The inverse projection model is specified by **P** = *γ***F**^−1^ (**p**), in which *γ* is an unknown scalar, meaning that **P** lies on a ray determined by **p**, but its distance stays unknown. Now, we consider two camera frames *C*_1_ and *C*_2_. In the first frame, the plane on which all the features lie is defined by {**N**_*C*_1__, *d*_*C*_1__}. Then, pair-wise corresponding image features **p**_*C*_1__ and **p**_*C_2_*_ satisfy
  (4) $${\text{p}}_{C_{2}}-\text{F}\left({\text{R}}_{C_{1}C_{2}}\frac{d_{C_{1}}{\text{F}}^{-1}({\text{p}}_{C_{1}})}{{\text{N}}_{C_{1}}^{T}{\text{F}}^{-1}({\text{p}}_{C_{1}})}+{\text{T}}_{C_{1}C_{2}}\right)=0$$
  Note that this constraint is also known as the homography constraint [28] when **F** is a pinhole camera projection model.
- **Motion constraint:** When a robot platform moves from one location to another, the translation of the camera and that of the LIDAR are equal to each other, as the sensors are fixed rigidly. Hence, we have
  (5) $${\text{T}}_{WC_{1}}-{\text{T}}_{WC_{2}}={\text{R}}_{WC}{\text{R}}_{WL}^{-1}\left({\text{T}}_{WL_{1}}-{\text{T}}_{WL_{2}}\right)$$

## 3. Algorithm Description

In order to estimate the relative transformation between a 3D LIDAR and a camera, we capture *N* (*N* ≥ 2) observations of a trihedron by both sensors. The sensors are individually calibrated in each configuration first to get their extrinsic parameters with respect to the world reference. Then, the LIDAR-camera extrinsic calibration is formulated as a nonlinear least squares problem in terms of the constraints introduced above. It is further solved by the Levenberg-Marquardt (LM) method [11] after properly estimating the initializations. An overview of the entire calibration procedure is presented in Algorithm 1. The details are subsequently introduced below.


**Algorithm 1:** 3D LIDAR-camera extrinsic calibration procedure.
**Input:**
*N* LIDAR-camera observations of a trihedron Manually selected regions of the trihedron's planes in the observations.**Output: R***_LC_* and **T***_LC_***for**
*i* = 1 → *N***do** **for**
*j* = 1 → 3 **do**  Estimate the *j^th^* plane 
$\left\{{\text{N}}_{L}^{j},d_{L_{i}}^{j}\right\}$ w.r.t the *i^th^* LIDAR frame; **end** Estimate the transformation {**R***_WL_i__*, **T***_WL_i__*};**end**Refine 
$\left\{{\text{N}}_{L_{i}}^{j},d_{L_{i}}^{j},{\text{R}}_{WL_{i}},{\text{T}}_{WL_{i}}\right\}$ based on all observations;Detect features on the first image;**for**
*i* = 2 → *N***do** Detect and match features on the *i^th^* image; Estimate the transformation {**R**_*C*_1_*C_i_*_, **T**_*C*_1_*C_i_*_}; Estimate 
$\left\{{\text{N}}_{C_{1}}^{j},d_{C_{1}}^{j},{\text{N}}_{C_{i}}^{j},d_{C_{i}}^{j}\right\}$; Estimate the transformation {**R**_*WC*_1__, **T**_*WC*_1__, **R**_*WC_i_*_, **T**_*WC_i_*_};**end**Initialize **R***_LC_* and **T***_LC_*;Refine the estimates of **R***_LC_* and **T***_LC_*.


### 3.1. 3D LIDAR Extrinsic Calibration

Given the *i^th^* LIDAR observation, this step is to estimate the transformation, **R***_WL_i__* and **T***_WL_i__*, from the world to the *i^th^* LIDAR frame. To this end, we first estimate the trihedron's planes according to the LIDAR observation. When a user specifies a set of 3D points that mostly lie on the trihedron's *j^th^* plane, the plane's parameters $\left\{{\text{N}}_{L_{i}}^{j},d_{L_{i}}^{j}\right\}$ are estimated by minimizing the following linear least squares problem:

(6) $$\arg\underset{{\text{N}}_{L_{i}}^{j},d_{L_{i}}^{j}}{\min}\sum_{k=1}^{M(i,j)}{\left\|{{\text{N}}_{L_{i}}^{j}}^{T}P_{L_{i}}^{j,k}-d_{L_{i}}^{j}\right\|}^{2}$$

where *M*(*i*, *j*) is the number of points on the plane. Once the three planes are determined with respect to the *i^th^* LIDAR frame, **R***_WL_i__* and **T***_WL_i__* are computed according to the trihedral constraint given in Equations (1) and (2).

When more than one observation is available, we can further refine the results by using the planarity constraints established between each pair of the LIDAR frames. Thus, we get

(7) $$\begin{array}{r} \arg\underset{{\text{N}}_{L_{1}}^{j},d_{L_{1}}^{j},{\text{N}}_{L_{i}}^{j},d_{L_{i}}^{j}}{\min}\sum_{i=2}^{N}\sum_{k=1}^{M(i,j)}{\left\|{{\text{N}}_{L_{1}}^{j}}^{T}\left({\text{R}}_{L_{i}L_{1}}{\text{P}}_{L_{i}}^{j,k}+{\text{T}}_{L_{i},L_{1}}\right)-d_{L_{1}}^{j}\right\|}^{2}\\ +\sum_{i=2}^{N}\sum_{k=1}^{M(1,j)}{\left\|{{\text{N}}_{L_{i}}^{j}}^{T}\left({\text{R}}_{L_{1}L_{i}}{\text{P}}_{L_{1}}^{j,k}+{\text{T}}_{L_{1}L_{i}}\right)-d_{L_{i}}^{j}\right\|}^{2} \end{array}$$

It is obtained by forming the first LIDAR frame with each of the remaining frames as pairs. Since **R**_*L_i_L*_1__, **T**_*L_i_L*_1__, **R**_*L*_1_*L_i_*_, and **T**_*L*_1_*L_i_*_ are the functions of the planes' parameters, Equation (7) is a nonlinear optimization problem with respect to the planes' parameters. This problem takes the previously estimated results as initializations and is solved by LM.

Let $\theta =\left\{{\text{N}}_{L_{1}}^{j},d_{L_{1}}^{j},{\text{N}}_{L_{i}}^{j},d_{L_{i}}^{j}\right\}$ be the parameters estimated in Equation (7), and *f* be the function that is optimized. Then, the Levenberg-Marquardt method starts from a given initial guess *θ*_0_ and iteratively updates the parameters via

(8) $$\theta_{t+1}=\theta_{t}+\Delta \theta_{t}$$

where Δ*θ_t_* is obtained by solving the following equation

(9) $$\left({\text{J}}^{T}\text{J}+\lambda \text{diag}\left({\text{J}}^{T}\text{J}\right)\right)\Delta \theta_{t}={\text{J}}^{T}\left(-f\left(\theta_{t}\right)\right)$$

Here *λ* is a damping parameter determined adaptively and **J** is the Jacobian matrix which is obtained conveniently by symbolic computation in MATLAB.

### 3.2. Camera Extrinsic Calibration

This step is to determine the transformations, {**R***_WC_i__*, **T***_WC_i__*}, from the world to the camera frames, together with the planes $\left\{{\text{N}}_{C_{i}}^{j},d_{C_{i}}^{j}\right\}$. In contrast to the LIDAR sensor, it is incapable of recovering all parameters from one image since no metric information is available. Hence, two LIDAR-camera observations are needed.

Given two LIDAR-camera observations, we first estimate **R**_*C*_1_*C*_2__ and **T**_*C*_1_*C*_2__ between the two camera frames. Once a user delimits the regions of the planes on two images, a set of point features are detected by SIFT [29] within the regions in the first image and then matched to the correspondences in the second one. The two sets of features are represented by $\left\{{\text{p}}_{C_{1}}^{k}\right\}$ and $\left\{{\text{p}}_{C_{2}}^{k}\right\}$, which satisfy the epipolar constraint [28]

(10) $${\text{F}}^{-1}{\left({\text{p}}_{C_{1}}^{k}\right)}^{T}{\text{EF}}^{-1}\left({\text{p}}_{C_{2}}^{k}\right)=0$$

Here, **E** is the essential matrix. The estimation of **E** and the recovery of **R**_*C*_1_*C*_2__ and **T**_*C*_1_*C*_2__ from **E** are the fundamental problems in computer vision, which are solved by the well-known eight-point algorithm [28]. However, the recovered **T**_*C*_1_*C*_2__ is of unit norm. We hence use the motion constraint defined in Equation (5) to get its scale.

Once the relative motion between two views is determined, we are able to determine the planes by taking advantage of the planarity constraint established between two images, as defined in Equation (4). Hence, in the presence of noise, we estimate the pose of a plane by

(11) $$\begin{array}{r} \arg\underset{\begin{array}{c} {\text{N}}_{C_{1}},d_{C_{1}}\\ {\text{N}}_{C_{2}},d_{C_{2}}\\ {\text{R}}_{C_{1}C_{2}},{\text{T}}_{C_{1}C_{2}} \end{array}}{\min}\sum_{k=1}^{M}{\left\|{\text{p}}_{C_{2}}^{k}-\text{F}\left({\text{R}}_{C_{1}C_{2}}\frac{d_{C_{1}}{\text{F}}^{-1}\left({\text{p}}_{C_{1}}^{k}\right)}{{\text{N}}_{C_{1}}^{T}{\text{F}}^{-1}\left({\text{p}}_{C_{1}}^{k}\right)}+{\text{T}}_{C_{1}C_{2}}\right)\right\|}^{2}\\ +{\left\|{\text{p}}_{C_{1}}^{k}-\text{F}\left({\text{R}}_{C_{2}C_{1}}\frac{d_{C_{2}}{\text{F}}^{-1}\left({\text{p}}_{C_{2}}^{k}\right)}{{\text{N}}_{C_{2}}^{T}{\text{F}}^{-1}\left({\text{p}}_{C_{2}}^{k}\right)}+{\text{T}}_{C_{2}C_{1}}\right)\right\|}^{2} \end{array}$$

in which **R**_*C*_1_*C*_2__, **T**_*C*_1_*C*_2__ are also refined. It is a nonlinear least squares problem solved by LM. The estimates of **N***_C_i__*. and *d_C_i__* are simply initialized with the corresponding parameters in the LIDAR frames for the simplicity. It is reasonable considering that the relative transformation between the two sensors is small when compared with those to the trihedron.

### 3.3. 3D LIDAR-Camera Extrinsic Calibration

With the above-estimated parameters, we now formulate the LIDAR-camera extrinsic calibration task as a nonlinear least squares problem. In terms of the planarity constraints established between the LIDAR and the camera frames, we get the form

(12) $$\arg\underset{{\text{R}}_{LC},{\text{T}}_{LC}}{\min}\sum_{i=1}^{N}\sum_{j=1}^{3}\sum_{k=1}^{M(i,j)}{\left\|{{\text{N}}_{c_{i}}^{j}}^{T}\left({\text{R}}_{LC}{\text{P}}_{L_{i}}^{j,k}+{\text{T}}_{LC}\right)-d_{c_{i}}^{j}\right\|}^{2}$$

It is solved by LM with the initializations obtained from

(13) $$\{\begin{array}{ll} {\text{R}}_{\text{LC}} & ={\text{R}}_{{\text{WC}}_{i}}{\text{R}}_{{\text{WL}}_{i}}^{-1}\\ {\text{T}}_{\text{LC}} & ={\text{T}}_{{\text{WC}}_{i}}-{\text{R}}_{{\text{WC}}_{i}}{\text{R}}_{{\text{WL}}_{i}}^{-1}{\text{T}}_{{\text{WL}}_{i}} \end{array}$$

with any *i* = 1 ⋯ *N*.

## 4. Experiments

We implement the proposed method in MATLAB. The running time of our algorithm is coarsely measured on a laptop with an Intel Core2Duo 2.26 GHz processor and 3 GB memory. Except for the manual input procedure, it takes about 20 seconds in average to perform the entire calibration when 9 LIDAR-camera observations are considered. Each contains 5,000 LIDAR points and 100 registered image points. In order to evaluate the proposed method, a series of experiments have been carried out. The algorithm is first tested on simulated data to validate its correctness and explore its sensitivity with respect to noise. Then, it is used to calibrate a real system composed of a 3D LIDAR and a camera. The calibration results are subsequently used for 3D reconstruction.

### 4.1. Simulations

The first experiment validates the correctness and numerical stability of our algorithm. We hereby generate sets of data to simulate multiple observations of a trihedron obtained by a 3D LIDAR-camera system. The system is of the following properties. The rotation and translation from the LIDAR to the camera are set, respectively, as *E*(**R***_LC_*) = [11.46°, 5.73°, 85.94°]*^T^* and **T***_LC_* = [0.4,−0.08, 0.2]*^T^* m, where *E*(**R***_LC_*) is the Euler angle of **R***_LC_*. An unorthogonal trihedron is synthesized, whose planes are defined by ${\text{N}}_{C_{1}}^{1}={\left[-0.342,0.937,0.067\right]}^{T}$, $d_{C_{1}}^{1}=-3.837\text{m}$, ${\text{N}}_{C_{1}}^{2}={\left[-0.325,-0.930,0.171\right]}^{T}$, $d_{C_{1}}^{2}=-7.710\text{m}$ and ${\text{N}}_{C_{1}}^{3}={\left[0.181,0.028,0.983\right]}^{T}$, $d_{C_{1}}^{3}=-2.466\text{m}$, with respect to the first camera frame. Each plane contains 5,000 LIDAR points and 100 registered image points. The simulated camera uses the following Mercator projection model:

(14) $$\{\begin{array}{ll} u & =(180-\text{atan}2(Y,X))M/360\\ \upsilon & =\text{acos}(Z/\sqrt{X^{2}+Y^{2}+Z^{2}})N/180 \end{array}$$

where (*u*, *υ*) denotes a pixel, and *M* × *N* = 1024 × 1024 is the resolution of an image. This projection model is one of the models that the Ladybug3 [25] camera has.

#### 4.1.1. Performance w.r.t. the Number of Observations

**Figure 2.**
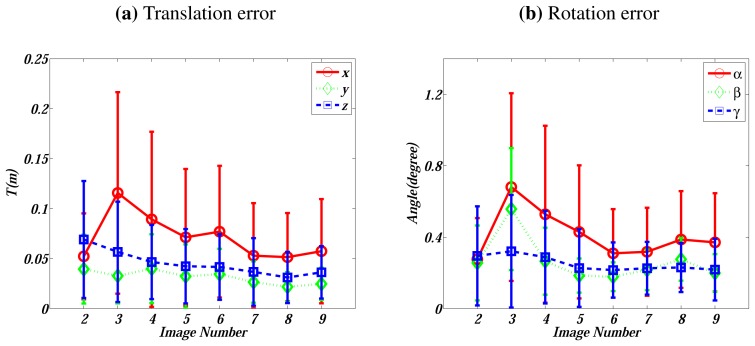
Errors *vs.* the number of observations. (**a**) presents the translation's absolute errors in *X*, *Y*, and *Z* directions. (**b**) shows the displaced Euler angle [*α*, *β*, *γ*]*^T^*.

The extrinsic calibration can be conducted with two or more LIDAR-camera observations. In this experiment, we investigate the impact of the observation number on the calibration performance. Nine LIDAR-camera observation pairs are generated, in which Gaussian noise with zero mean and *σ* standard deviation is added. We randomly select *σ* from the range of [0, 0.2] *m* for LIDAR points and from the range of [0, 1] pixels for image features. We vary the number of observations from 2 to 9. For each number, 200 independent trials are carried out. The estimated parameters **R***_LC_* and **T***_LC_* in each trial are compared with the ground truth and measured, respectively, by the displaced Euler angle of the rotation and the absolute error of the translation. Figure 2 plots the mean and standard deviation of the errors.

Figure 2 shows no obvious benefits achieved when the number of observations increases. The reason is that, even when we use two observations, in total there are already six planes taken into consideration. In our simulations, there is even a peak on the error corresponding to 3 observations, partly because the impact of noise is larger than the benefit achieved from the increase of observations. Hence, on the leverage of complexity and performance, throughout all the following experiments, we continue using two LIDAR–camera observations.

#### 4.1.2. Performance w.r.t. the Noise on LIDAR Points

**Figure 3.**
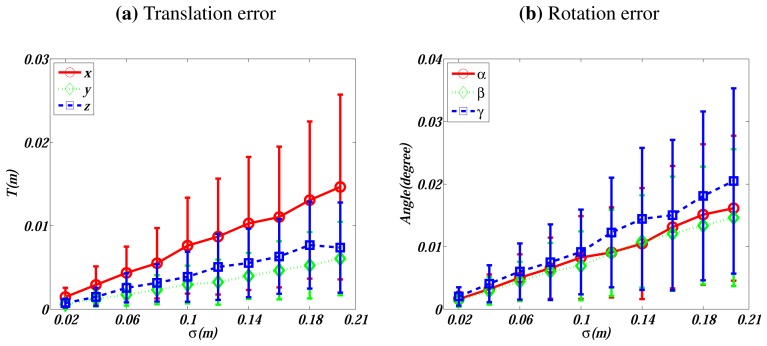
Errors *vs*. the noise level on LIDAR points. (**a**) presents the translation's absolute errors in *X*, *Y*, and *Z* directions. (**b**) shows the displaced Euler angle [*α*, *β*, *γ*]*^T^*.

Real ranging sensors produce noisy measurements. Hence, this experiment explores the sensitivity with respect to noise on LIDAR points. We conduct the experiment on the first two simulated observations. Zero mean Gaussian noise is added to points of the LIDAR observations, with *σ* varying from 0.02 to 0.2 m. Analogous to the previous case, we conduct 200 independent trials for each noise level. The errors on the translation and rotation are evaluated and plotted in Figure 3.

Figure 3 shows that the errors increase linearly with the noise level. When *σ* = 0.1 m, which is a noise level of a practical LIDAR, the translation errors are around 0.005 m in *Y* and *Z* directions, and 0.01 m in *X* direction. The rotation errors are about 0.01°. In our simulated configurations, *X* represents the direction of the optical axis, along which depth information degenerates so that larger errors are resulted in [30].

#### 4.1.3. Performance w.r.t. the Noise on Image Points

**Figure 4.**
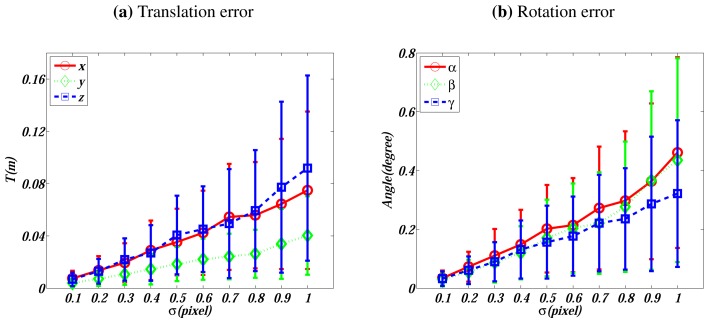
Errors *vs.* the noise level on image points. (**a**) presents the translation's absolute errors in *X*, *Y*, and *Z* directions. (**b**) is the displaced Euler angle [*α*, *β*, *γ*]*^T^*.

The feature detection and matching algorithm we use in this work is SIFT [29], which is of sub-pixel accuracy. In this experiment, we investigate the sensitivity with respect to the noise on matched image features. Zero mean Gaussian noise with *σ* ∈ [0.1, 1] pixels is added to each feature point on the first two simulated image data. Analogously to above cases, 200 trials are conducted for each noise level. The performance is evaluated and plotted in Figure 4.

Figure 4 also presents a linear relationship between the errors and the noise level. When *σ* = 0.5 pixels, which is a noise level higher than the normal noise, the translation errors are smaller than 0.04 m and the rotation errors are around 0.2°.

### 4.2. Real Data

**Figure 5.**
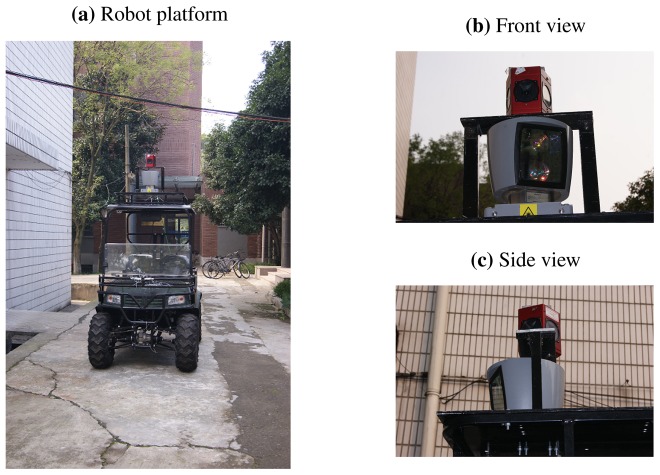
The robotic platform and the sensors in our experiment. (**a**) is the robotic platform, which is equipped with a Ladybug3 camera and a 3D Velodyne HDL-64E LIDAR. (**b**) shows the front view of the two sensors, and (**c**) is the side view.

To further evaluate the proposed algorithm, we employ it to calibrate a real system and use the calibration results to reconstruct 3D scenes. The system is composed of a 3D Velodyne HDL-64E LIDAR [26] and a Ladybug3 spherical vision system [25], which are rigidly mounted on the roof of a vehicle, as shown in Figure 5. Both sensors produce omnidirectional measurements.

**Figure 6.**
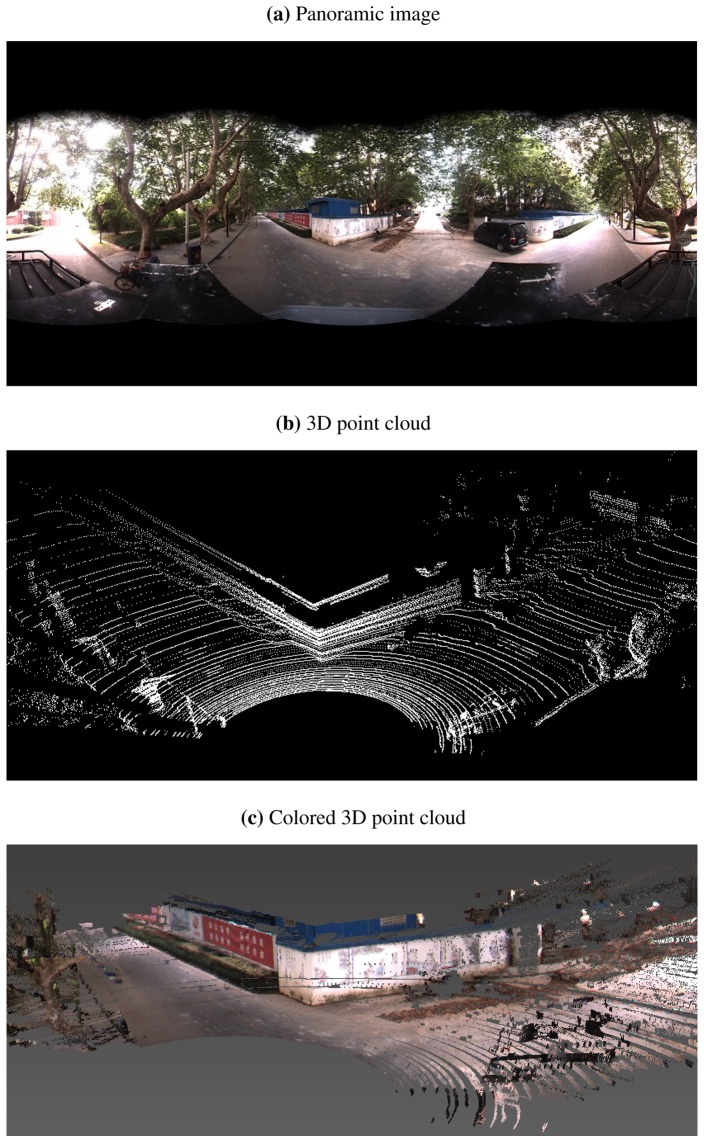
The first LIDAR-camera view used for calibration. (**a**) is the panoramic image captured by a Ladybug3 camera, and (**b**) is the 3D point cloud collected by a Velodyne HDL-64E LIDAR. (**c**) shows the reconstructed 3D scene.

**Figure 7.**
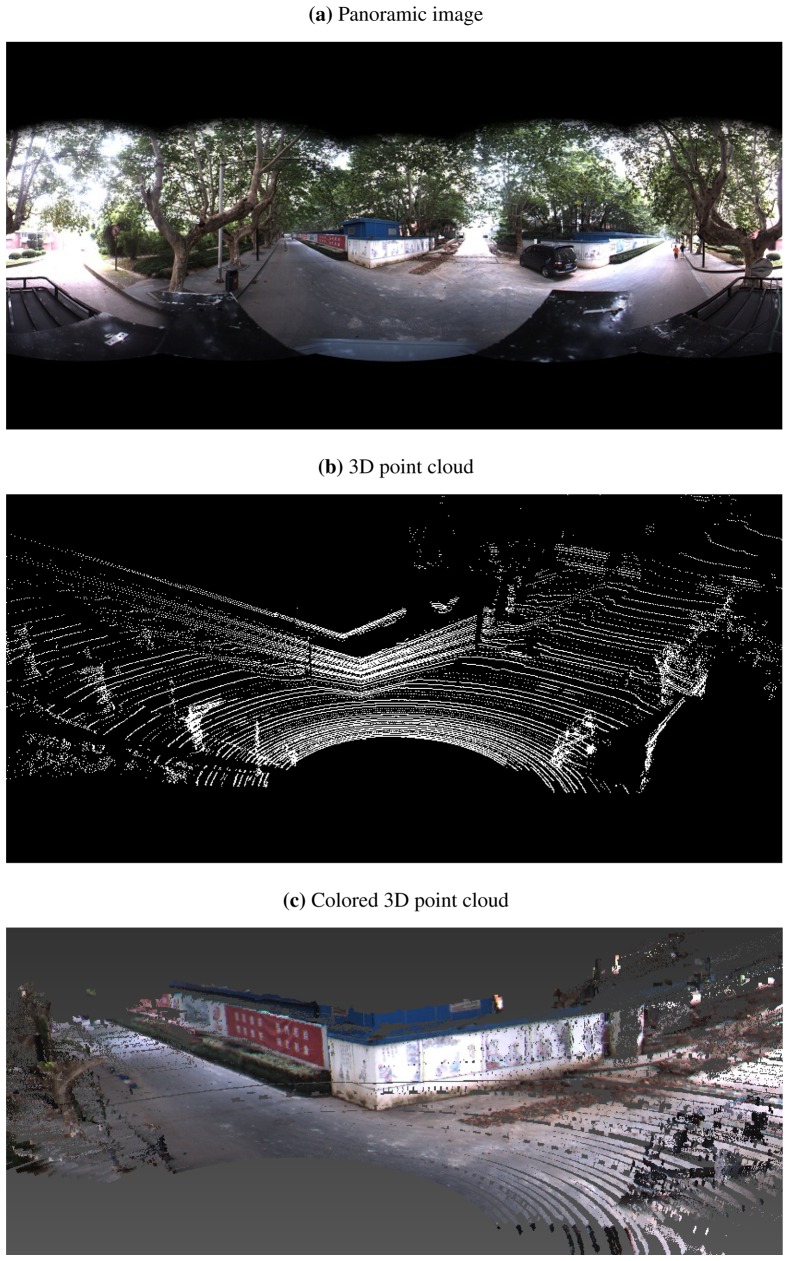
The second LIDAR-camera view used for calibration. (**a**) is the panoramic image captured by a Ladybug3 camera, and (**b**) is the 3D point cloud collected by a Velodyne HDL-64E LIDAR. (**c**) shows the reconstructed 3D scene.

**Figure 8.**
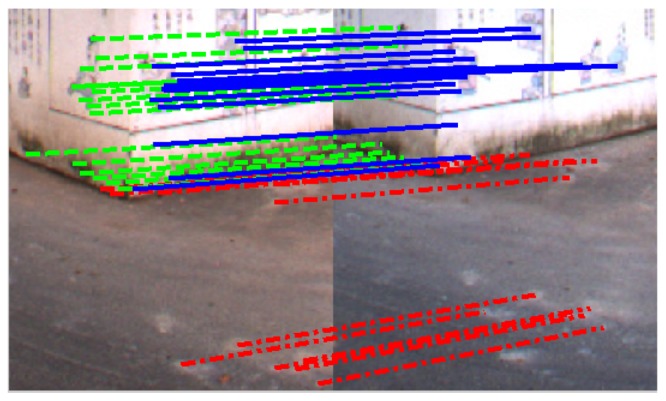
A portion of matched features on the trihedron. The matched feature pairs on the three planes are marked with lines in different styles.

In the experiments, we collect two LIDAR-camera measurements of a scenario containing a trihedral object. The trihedron consists of two adjacent walls of a building, together with the ground plane, as shown in Figures 6 and 7. Due to imperfect construction techniques and noise, the planes of the trihedron are not strictly orthogonal to each other. During the calibration procedure, regions of the planes are manually marked out on both LIDAR and image data, and features on the imaged trihedrons are detected and matched by SIFT [29]. A portion of the matched feature points are shown in Figure 8. Table 1 lists the calibration results of our method. For the purpose of comparison, we also include the results obtained by the checkerboard pattern-based method [12] using six observations. From the results, it is difficult to determine which one is more accurate, since no ground truth is available. Our proposed method, however, is more convenient, as it is easier to collect calibration data and requires less manual input.

In order to validate the calibration results, the determined extrinsic parameters are further used for 3D reconstruction. With the calibrated **R***_LC_* and **T***_LC_*, 3D LIDAR points in a view are first transformed into the camera frame and then registered to the image. The colors of registered image pixels are taken to render the corresponding upsampled LIDAR points. Figure 6(c) and Figure 7(c) demonstrate the colored 3D scenes of the two calibration views (only the data within a 180° field of view are shown for a better visibility), from which we see that the walls and the bushes are reconstructed well.

## 5. Conclusions

In this paper, we have presented a new method of conducting the extrinsic calibration for a 3D LIDAR-camera system. Specifically, instead of using planar checkerboard patterns, we take advantage of arbitrary trihedral objects, which might be either orthogonal or not, for calibration. This kind of configuration is ubiquitous in structured environments, so that it is very convenient for a mobile robot to collect data. We have validated the algorithm on both simulated and real scenarios. Although the experimental results are presented from 3D LIDAR and omnidirectional camera systems, the algorithm is applicable to systems composed of any kind of 3D LIDARs and cameras. Our method is interesting for both indoor or outdoor mobile robots equipped with such sensors. The calibration results can be further used for data fusion applications.

## Figures and Tables

**Table 1.** Calibration results of a real 3D LIDAR-camera system.

	*T_X_* (m)	T*_Y_* (m)	*T_Z_* (m)	*α* (deg)	*β* (deg)	*γ* (deg)
The proposed method	0.257	0.007	−0.323	−1.788	1.446	−88.542
The method in [12]	0.203	0.036	−0.285	−1.358	1.799	−88.996
